# Aberrant NFATc1 signaling counteracts TGFβ-mediated growth arrest and apoptosis induction in pancreatic cancer progression

**DOI:** 10.1038/s41419-019-1682-2

**Published:** 2019-06-06

**Authors:** Marie C. Hasselluhn, Geske E. Schmidt, Volker Ellenrieder, Steven A. Johnsen, Elisabeth Hessmann

**Affiliations:** 1 Department of Gastroenterology and Gastrointestinal Oncology, University Medical Center, Goettingen, Germany; 2 Department of General, Visceral and Pediatric Surgery, University Medical Center, Goettingen, Germany; 3 Division of Gastroenterology and Hepatology, Department of Internal Medicine, Mayo Clinic, Rochester, MN USA

**Keywords:** Pancreatic cancer, Translational research

## Abstract

Given its aggressive tumor biology and its exceptional therapy resistance, pancreatic ductal adenocarcinoma (PDAC) remains a major challenge in cancer medicine and is characterized by a 5-year survival rate of <8%. At the cellular level, PDAC is largely driven by the activation of signaling pathways that eventually converge in altered, tumor-promoting transcription programs. In this study, we sought to determine the interplay between transforming growth factor β (TGFβ) signaling and activation of the inflammatory transcription factor nuclear factor of activated T cells (NFATc1) in the regulation of transcriptional programs throughout PDAC progression. Genome-wide transcriptome analysis and functional studies performed in primary PDAC cells and transgenic mice linked nuclear NFATc1 expression with pro-proliferative and anti-apoptotic gene signatures. Consistently, NFATc1 depletion resulted in downregulation of target genes associated with poor PDAC outcome and delayed pancreatic carcinogenesis in vivo. In contrast to previous reports and consistent with a concept of retained tumor suppressive TGFβ activity, even in established PDAC, TGFβ treatment reduced PDAC cell proliferation and promoted apoptosis even in the presence of oncogenic NFATc1. However, combined TGFβ treatment and NFATc1 depletion resulted in a tremendous abrogation of tumor-promoting gene signatures and functions. Chromatin studies implied that TGFβ-dependent regulators compete with NFATc1 for the transcriptional control of jointly regulated target genes associated with an unfavorable PDAC prognosis. Together, our findings suggest opposing consequences of TGFβ and NFATc1 activity in the regulation of pro-tumorigenic transcription programs in PDAC and emphasize the strong context-dependency of key transcription programs in the progression of this devastating disease.

## Introduction

Transforming growth factor β (TGFβ) is a cytokine with pivotal regulatory functions during development and in tissue homeostasis^1^. Physiologically, TGFβ binding to its receptors leads to phosphorylation of the receptor-regulated Smads (Smad2, Smad3), which subsequently form complexes with Smad4 and translocate to the nucleus where they regulate gene transcription^2–4^. In accordance with its critical involvement in key biological processes in nearly every tissue type^5,6^, dysregulation of TGFβ signaling can have severe consequences on cellular integrity and may foster neoplastic transformation and tumorigenesis^7^. Importantly, the output of TGFβ signaling during carcinogenesis and cancer progression is highly complex and ambivalent. In untransformed cells and in early carcinogenesis, TGFβ induces cell cycle arrest and blocks incipient tumors from further malignant progression^8–10^. However, malignant cells regularly circumvent or distort the tumor-suppressive influence of TGFβ signaling, thus driving tumor growth, invasion, and metastasis^1,11^.

The dual role of TGFβ in malignancy is particularly evident in pancreatic ductal adenocarcinoma (PDAC), a highly lethal disease with a 5-year survival rate <8%^12^. TGFβ is strongly expressed in both the epithelial and stromal compartment of PDAC and dysregulation of TGFβ signaling is one of the most frequently molecular disturbances characterizing this highly heterogeneous disease^13^. The opposing roles of TGFβ in PDAC development and progression have been primarily attributed to the mutational or functional inactivation of the TGFβ-controlled Smad pathway. Indeed, genetic inactivation of the *Smad4* tumor-suppressor gene constitutes a frequent event in pancreatic carcinogenesis^13,14^ and is closely related to reduced tumor-suppressive activity of TGFβ^15–17^. However, loss of TGFβ-mediated reduction of tumor growth can also occur independently of genetic *Smad4* inactivation, for instance, by sequential activation of nuclear factor of activated T cells (NFAT) and Myc transcription factors (TFs)^18^. NFAT proteins belong to a family of inflammatory TFs that have been primarily described in the context of T cell activation^19^. However, NFAT activity is not restricted to the immune system but is involved in the development and progression of several malignancies^20–23^. Accordingly, we have previously demonstrated a pivotal role of NFATc1 in pancreatic carcinogenesis and PDAC progression where the TF strongly promotes tumor cell proliferation, invasion, metastasis, and acquisition of stemness features in vitro and in vivo^24–26^. Strikingly, and in line with its strong oncogenic potential, NFAT TFs have been characterized as critical mediators of the TGFβ switch in PDAC cells. Upon TGFβ-induced expression, NFATc1 accumulates in the nucleus and displaces the Smad3 repressor complex from the TGFβ-inhibitory element of the *Myc* promoter, thus driving expression of the Myc oncogene. Importantly, genetic or pharmacological inhibition of NFATc1 activity reduces *Myc* transcription and cell proliferation and partially restores TGFβ-mediated growth suppression^18^.

Despite the intriguing characterization of NFATc1 as a determinant of the opposing TGFβ functions in the pancreas, the extent to which NFATc1 determines the transcriptome-wide effects of TGFβ signaling in PDAC remain largely elusive. In this work, we sought to build on our previous findings characterizing NFATc1 as a mediator of oncogenic TGFβ activity and performed transcriptome-wide analyses as well as functional studies in primary PDAC cells and transgenic mice to further elucidate the complex interplay of TGFβ and NFATc1 in PDAC progression.

## Material and methods

### Mouse strains and primary tumor cells

*P48-Cre*, *pdx-Cre*, *Kras*^*G12D*^, and *NKC p48* mice have been previously described^24,27,28^. *NFATc1*^*fl/fl*^ mice were kindly provided by Laurie Glimcher^29^ and are already described in the PDAC context^24,25^. All mice were genotyped by polymerase chain reaction (PCR), as described previously^24,30^. All procedures were carried out using protocols approved by the Institutional Animal Care and Use Committee at the University Medical Center Goettingen.

NKC-II, NKC-VI, and NKC-VIII primary pancreatic tumor cells were extracted from three different tumor-bearing *NKC p48* mice, respectively, using a protocol already mentioned elsewhere^24^.

### Cultivation of cells, TGFβ treatment, and transfection

Primary tumor cells were cultivated using Dulbecco’s modified Eagle’s medium containing 4.5 g/L d-Glucose, l-Glutamine, supplemented with 10% fetal bovine serum, and 1% nonessential amino acids. Prior to TGFβ1 treatment (Peprotech, 100-21, 10 ng/ml), cells were synchronized by serum starvation for 24 h.

Cells were transfected with small interfering RNA (siRNA) from Thermo Scientific (siNFATc1 #1 288360; siNFATc1 #2 MSS275982) and siLentFect lipid reagent (BioRad) in 250 µl OptiMEM (Gibco, 31985-062) per 6-well to introduce the NFATc1 knockdown in parallel to serum starvation.

The sequences of used siRNAs are the following:

siNFATc1 #1: 5′-GCG UUU CAC GUA CCU UCC Utt-3′;

siNFATc1 #2: 5′-AGG ACA GGA AGU AUC CCG AAG GCC C-3′.

### Wound healing assay

Cells were seeded confluent in triplicates in a 12-well plate. After 8 h, cells were serum starved overnight (o/n). One wound per well was introduced with a 10-µl tip prior to live cell imaging for 12 h (one image/h). Migration speed was determined by measuring cell border distances in triplicates on each image by Fiji^31^. The migration speed of TGFβ-treated samples was normalized to control sample (serum-free only).

### Invasion assay

To measure invasion, a two-chamber assay was performed. To this end, 40,000 cells were seeded in 50% Matrigel in collagen-coated inserts for 24-well plates (BD, 353097, 8 µm pore size). Serum-free medium ± TGFβ was added to the chamber, while fetal bovine serum-containing growth medium was provided in the wells. Matrigel was removed after 48 h incubation, cells were fixed by 20 min 4% paraformaldehyde (PFA) in phosphate-buffered saline (PBS) at room temperature (RT) prior to 4,6-diamidino-2-phenylindole (DAPI) staining (Sigma-Aldrich, D9542; 1:2000) and mounting (Thermo Scientific, 9990402).

### Bromodeoxyuridine (BrdU)

5,000 cells of NKC-II, NKC-VI, and NKC-VIII were seeded in a 96-well plate in quadruplicates per condition. Serum starvation and NFATc1 knockdown was conducted the next day. After 24 h of knockdown, TGFβ was added to the respective wells. After 42 h of NFATc1 knockdown, BrdU labeling reagent was added to each well for 6 h (Roche, 11647229001). Subsequently, the supernatant was discarded and plates stored at 4 °C o/n. Further steps were performed according to the manufacturer’s instructions. Background was deducted from measurements prior to normalization to control (serum-free) condition.

### Quantitative real-time PCR (qRT-PCR)

RNA isolation was performed as described below. The supernatant was aspirated, and cells were scraped in TRIzol (Qiagen) prior to phenol-chloroform purification. 1 µg RNA was reverse-transcribed by the iScript cDNA Synthesis Kit (BioRad, 170-8891). qRT-PCRs were performed in triplicates using iTaq Universal SYBR Green Supermix (BioRad, 172-5125) with StepOne Plus Real-Time PCR System (Applied Biosystems). Primer sequences used in this study are listed in Table S1. All targets were normalized to Rplp0 housekeeping gene before normalization to serum-free treatment control. For clear representation of results, we refrained from displaying the respective significances in each graph (GraphPad Prism, version 7.03). Minimal statistical significance of all experiments shown is *p* < 0.05 in siCtrl vs siNFATc1+TGFβ, recapitulating the extremes in unfavorable prognosis in PDAC dataset, if not stated otherwise.

### Immunohistochemistry (IHC), immunofluorescence, cell cycle analysis, and Annexin/propidium iodide (PI) staining

Hematoxylin and eosin staining and IHC were performed as previously described^30^. Utilized antibodies are listed in Table S2. Mouse pancreatic intraepithelial (PanIN) stages were classified according to histopathologic criteria as recommended elsewhere^28,32^.

For immunofluorescence, NKC-II, NKC-VI, and NKC-VIII cells were seeded on cover slips in 12-well plates. Cells were fixed by 20 min 4% PFA in PBS at RT prior to washing with PBS+0.4% Triton X-100. Subsequent to 1 h blocking with 10% normal goat serum in PBS+0.4% Triton X-100 at RT, Smad2/3 antibody (BD Biosciences, 610842; 1:100) was diluted in 2% normal goat serum in PBS+0.4% Triton X-100 and incubated o/n at 4 °C. Secondary antibody (Abcam, ab175473; 1:1000) was incubated for 1 h at RT prior to DAPI staining (Sigma-Aldrich, D9542; 1:2000) and mounting (Thermo Scientific, 9990402).

For cell cycle analysis and Annexin/PI staining, NKC cells were seeded in 6-wells and treated as described above. After 48 h NFATc1 knockdown and 24 h TGFβ treatment, cells were trypsinized and both supernatant and cell solution were subjected to flow cytometric analysis. Prior to cell cycle analysis, cells were fixated in 70% Ethanol for 30 min at 4 °C, treated with 5 µg/ml RNase A (Sigma-Aldrich, R4642), and stained with 50 ng/µl Hoechst (Sigma, B2261). For Annexin/PI staining, cells were centrifuged, resuspended in Annexin Binding Buffer (Biolegend, 422201), and stained with 3 µl Annexin V antibody (Biolegend, 640920) and 1 µl PI (Sigma-Aldrich, P4864). Single cells were measured by FACS Canto II (BD) using the FACS Diva Software (version 6.1.3), and data were analyzed by the FlowJo software (version 10.1r1) and visualized by GraphPad Prism.

### Western blot

Supernatant was aspirated and cells were washed with PBS before adding whole-cell lysis buffer to the each 6-well. Cells were scraped, incubated for 1 h on ice, and spun down prior to Bradford protein measurement. 20 µg of protein was applied for each sample on sodium dodecyl sulfate-polyacrylamide gel electrophoresis. Blotting was performed using TurboBlot system (BioRad, 10026938). After 1 h blocking in 5% milk powder in TBS+0.1% Tween20, protein membranes were incubated with their respective antibody dilutions at 4 °C o/n (see Table S3). Secondary antibodies were applied for 1 h at RT (1:10,000; CST, #7074, #7076) before detecting protein bands at Intas ECL chemocam imager using chemiluminescence (Perkin Elmer, 265-18121).

### Chromatin immunoprecipitation (ChIP)

ChIP analyses were performed as described previously^33^ with the following modifications: NKC cells were fixed in 1.5% formaldehyde in PBS for 20 min and quenched with 125 mM Glycine for 5 min at RT prior to harvest in Nelson buffer^34^. Subsequent to resuspension in Gomes buffer^35^, the chromatin extract was subjected to 30 cycles of sonication (30 s ON/OFF) using a Bioruptor Plus (Diagenode, UCD-300, v1.1). Preclear was performed using 100 µl 50% Sepharose A Beads (Millipore, 16-125) and respective shares were incubated with their primary antibodies o/n at 4 °C (see Table S4 for antibody details). The next day, after 2 h of incubation with 50 µl bovine serum albumin-blocked 50% Sepharose A Beads per condition, beads were washed as described and subsequently subjected to RNase A treatment (Sigma-Aldrich, R4642) and incubated with Proteinase K (Applichem, A4392) o/n at 65 °C. DNA was precipitated using 0.4 M LiCl and linear polyacrylamide (Bioron, 130101) and isolated using phenol–chloroform–isoamyl alcohol extraction. ChIP qRT-PCR was conducted in triplicates using materials described above and primers listed in Table S4. Results were analyzed by normalization to input and visualized by GraphPad Prism. The background was determined using IgG samples.

### RNA-seq

Subsequent to NFATc1 knockdown (Thermo Scientific, 288360; 48 h) under serum-free condition and TGFβ treatment for 24 h, cells were harvested for protein and RNA isolation (triplicates), respectively. Purity and integrity of RNA was checked on an agarose gel prior to cDNA synthesis. Western blot and qRT-PCRs confirmed successful NFATc1 knockdown and activation of TGFβ pathway.

Illumina kits (RS-122-2001; RS-122-2002) were used for library preparation according to the manufacturer’s instructions. cDNA concentrations were controlled by Qubit (Thermo Scientific, Q32854) and fragment sizes by Agilent 2100 bioanalyzer (Agilent, 5067-4626), respectively, prior to sequencing by Transcriptome and Genome Analysis Laboratory of the University Medical Center Goettingen, Germany.

After uploading the data to Galaxy web platform, the public server at usegalaxy.org^36^ was used to map Fastq files to the murine transcriptome (mm9) using TopHat2 tool^37^ (version 2.1.0) with very sensitive bowtie2 settings with subsequent sorting by SortSam (version 1.136.1). After read counting via HTSeq^38^ (union mode; version 0.9.1), principal component analysis (PCA) and sample-to-sample distances were performed to assess similarities between replicates. Their respective plots were calculated based on DESeq2^39,40^. Differential regulation and normalized fragment counts were obtained by Cufflink tools, Cuffdiff (version 2.2.1) and Cuffnorm (geometric; version 2.2.1.1), respectively^41,42^. Fragments Per Kilobase Million (FPKM) threshold >5 was established to reduce background, considering only 39.7% of the whole murine genome (9225/23,234 genes). Heatmaps were created by calculating *z* score of each replicate. Means of each condition were processed using hierarchical clustering by Cluster 3.0 (Euclidean distance; version 1.54) in combination with Java TreeView (version 1.1.6r4).

### Gene ontology (GO) and The Cancer Genome Atlas (TCGA)

GO^43,44^ was used to determine the function of leading edge-regulated genes selected from unfavorable prognostic genes in PDAC dataset of the human protein atlas^45,46^. By using the overrepresentation test of Protein Analysis Through Evolutionary Relationships (PANTHER; version 13.1) with Fisher’s exact test with false discovery rate (FDR) <0.05 multiple test correction, the function of those proteins was assessed by classification with the PANTHER GO Slim Biological Process algorithms^47,48^.

TCGA data^49^ was searched for NFATc1 target gene-related survival with OncoLnc tool^50^ by setting percentiles at 50 (patient groups *n* = 87).

### Gene set enrichment analysis (GSEA)

GSEA^51,52^ was performed with Signal2Noise metric for ranking genes and significantly enriched pathways are shown (FDR ≤ 0.25). For the Normal-Tumor-Stroma dataset^53^, log2fold change of ≥0.5 and ≤−0.5 was set to differentiate the respective groups.

### Statistical analyses

Data are presented as mean ± standard deviation (SD). Significance was tested by Student’s *t* test, designating significance as *p < 0.05, **p < 0.01, and ***p < 0.001.

## Results

### TGFβ signaling is active in transgenic mice and primary PDAC cells with constitutive NFATc1 expression

**Fig. 1:**
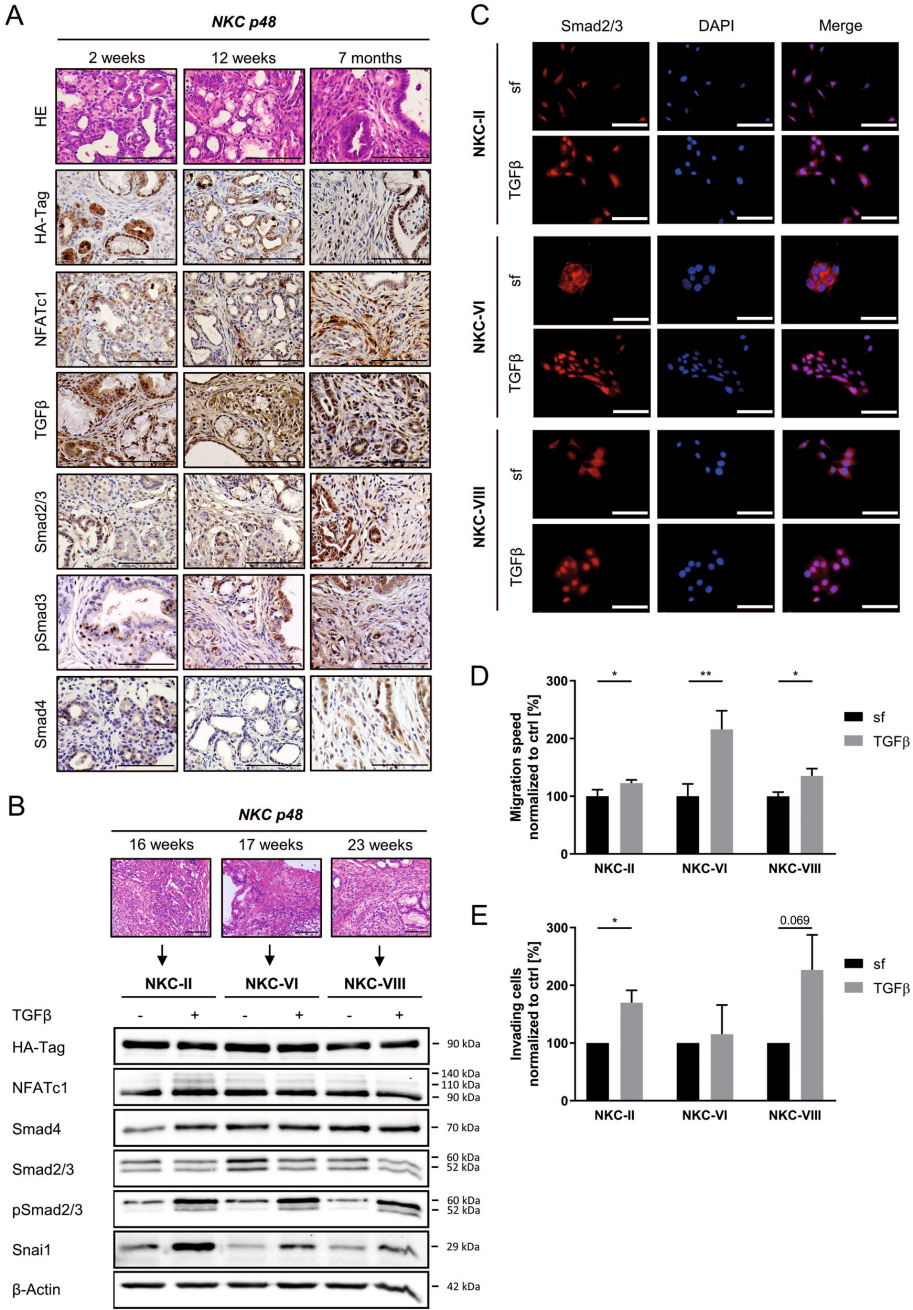
Transforming growth factor β (TGFβ) signaling is active in transgenic mice and primary pancreatic ductal adenocarcinoma (PDAC) cells with constitutive NFATc1 expression. **a** Hematoxylin and eosin (HE) and immunohistochemical of *NKC p48* mice representing different age groups corresponding to different stages of PDAC development (×400, scale bar: 100 µm). **b** HE stainings (×200, scale bar: 100 µm) certify established PDAC of three individual *NKC p48* mice. Western blot analysis of the indicated proteins upon TGFβ treatment (10 ng/ml) in primary PDAC cells derived from respective *NKC p48* mice (NKC cells). **c** Immunofluorescence demonstrating TGFβ-mediated translocation of receptor Smads (Smad2/3) into nuclei of primary PDAC cells (×400, scale bar: 25 µm). **d** Wound healing assay (*n* = 4) and **e** invasion assay in the presence and absence of TGFβ (*n* = 3)

In order to study the functional and mechanistic consequences of the TGFβ-NFATc1 interplay in transcriptional regulation in PDAC, we took advantage of a previously described genetically engineered mouse model (GEMM), which expresses constitutive nuclear HA-tagged NFATc1 along with oncogenic Kras under the control of a pancreas-specific recombinase (*cnNFATc1;Kras*^*G12D*^*;p48-Cre*, further designated as *NKC p48*)^24^. Constitutively active NFATc1 in this model dramatically accelerates pancreatic carcinogenesis with metaplastic lesions and low-grade PanIN PDAC precursor lesions already occurring 2 weeks postnatal (Fig. 1a). 12-week-old mice already displayed a full PanIN spectrum, while 7-month-old animals all suffer from PDAC^24^. To investigate whether TGFβ signaling is active throughout pancreatic carcinogenesis and PDAC progression in this model, we performed IHC analysis of TGFβ and observed its strong expression in both the cellular stroma compartment and epithelial cells (Fig. 1a). Importantly, TGFβ-responsive Smad proteins could be detected in both the cytoplasm and nucleus of pancreatic pre-neoplastic and tumor cells (Fig. 1a), suggesting that canonical TGFβ signaling is active in this model.

In order to further elucidate the functional and mechanistic consequences of TGFβ signaling in the context of constitutively active NFATc1 we isolated primary PDAC cells from three different tumor-bearing *NKC p48* mice (further designated as NKC-II, NKC-VI, and NKC-VIII) (Fig. 1b). While TGFβ treatment heavily increased Smad2/3 phosphorylation (Fig. 1b) and nuclear translocation (Fig. 1c), HA-NFATc1 levels remained unaltered upon treatment (Fig. 1b), confirming that the constitutive nuclear expression of the TF is not influenced upon TGFβ stimulation. Of note, all cells expressed comparable levels of Smad4 (Fig. 1b), indicating that this master tumor-suppressor gene was not deleted during PDAC development of the donor mice. In line with previous results^54^, TGFβ treatment significantly induced the expression of the epithelial-to-mesenchymal transition (EMT)-inducing TF Snai1 (Fig. 1b). Accordingly, TGFβ treatment accelerated the wound closure and invading capacities of primary NKC cells (Fig. 1d, e), confirming the susceptibility of these cells toward TGFβ-triggered migration and invasion.

Together, these results demonstrate intact TGFβ signaling in tissue of primary PDAC cells from *NKC p48* mice and hence illustrate the suitability of this model for studying TGFβ- and NFATc1-driven transcription in PDAC.

### NFATc1 loss restores anti-proliferative and pro-apoptotic gene signatures in response of TGFβ

In order to study the transcriptional consequences of TGFβ treatment and NFATc1 activity in PDAC cells, we performed RNA-seq analysis in the presence and absence of NFATc1 in either serum-starved or TGFβ-treated NKC-II cells, respectively (Fig. S1A). qRT-PCR analysis (Fig. S1B) and immunoblot (Fig. S1C) confirmed successful NFATc1 knockdown and TGFβ treatment in all three replicates of each condition. PCA and sample-to-sample distances confirmed the similarity of triplicates (Fig. S1D) and indicated four distinct treatment groups.

Aiming at the identification of TGFβ-dependent NFATc1 targets (further referred to as TGFβ dependency) as well as TGFβ effects that occur in an NFATc1-dependent manner (further designated as NFATc1 dependency), we compared TGFβ-treated and untreated samples in the presence and absence of NFATc1 (siCtrl vs siCtrl+TGFβ and siNFATc1 vs siNFATc1+TGFβ) as well as NFATc1-positive and -negative samples in the presence and absence of TGFβ (siCtrl vs siNFATc1 and siCtrl+TGFβ vs siNFATc1+TGFβ), respectively. In total, 5.9% of genes were regulated upon NFATc1 depletion and/or TGFβ treatment (544/9225 expressed genes; log2fold change ≥1; ≤−1, FPKM > 5). Consistent with previous findings associating NFATc1 expression with transcriptional activation^24^, NFATc1 knockdown resulted in a higher number of downregulated than upregulated genes, irrespective of TGFβ treatment (Fig. S1E, NFATc1 dependency). In contrast, TGFβ treatment in NFATc1-depleted cells resulted in a higher number of downregulated than upregulated genes compared to TGFβ treatment in the presence of NFATc1, where the number of downregulated and upregulated genes was nearly equal (Fig. S1E, TGFβ dependency), suggesting that TGFβ-mediated transcription at least partially relies on NFATc1 activity.

**Fig. 2:**
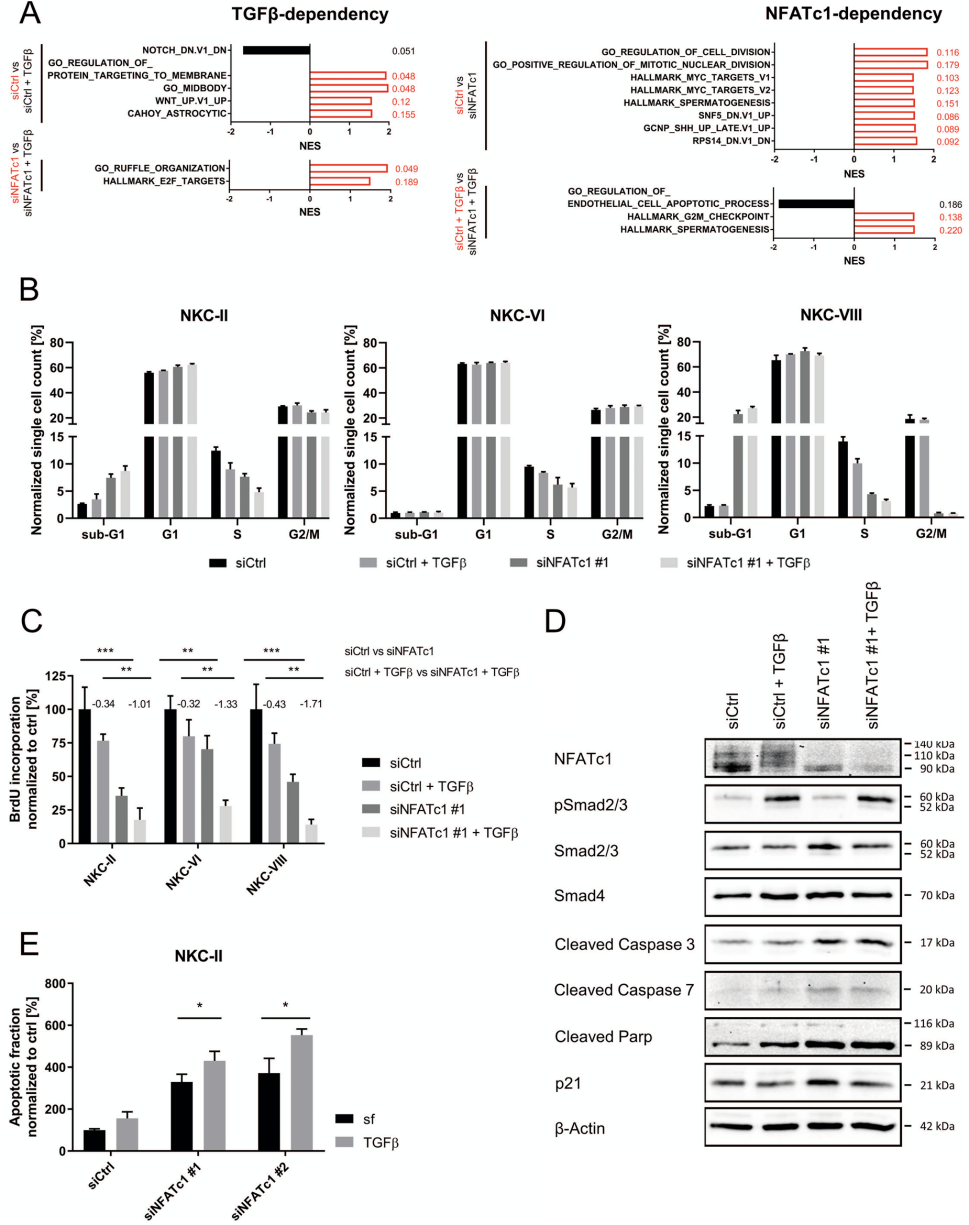
NFATc1 loss in the presence of transforming growth factor β (TGFβ) induces anti-proliferative and pro-apoptotic gene signatures. **a** Top differentially regulated datasets determined by gene set enrichment analysis (hallmarks, gene ontology, and oncogenic signatures) according to NFATc1 and TGFβ dependency. All significant hits with false discovery rate (FDR) ≤ 0.25 are considered (Fragments Per Kilobase Million >5, respective FDR values are displayed). **b** Cell cycle analysis classifying NKC cells according to corresponding phases (*n* = 3). **c** Bromodeoxyuridine assay in NKC cells determining proliferative capacity upon TGFβ treatment and NFATc1 knockdown, respectively (log2fold changes between TGFβ-treated and untreated samples are indicated; *n* = 3). **d** Western blot showing expression of cell cycle arrest- and apoptosis-related proteins in NKC-II cells. **e** Apoptosis induction was evaluated by flow cytometry subsequent to Annexin/propidium iodide staining (*n* = 3) in NKC-II cells

GSEA was performed to decipher differentially regulated gene signatures in the two aforementioned subgroups. In the TGFβ-dependency group, we primarily detected gene signatures related to proliferation, cytokine expression, or Wnt signaling, all of which were found to be positively enriched in unstimulated NFATc1-positive or -negative cells. In contrast, the majority of the top regulated gene signatures in the NFATc1-dependency group were associated with proliferation (Fig. 2a). Consequently, decreased DNA synthesis and abrogated tumor cell proliferation upon TGFβ treatment in primary NKC cells were enforced by additional NFATc1 knockdown (Fig. 2b, c). Moreover, GSEA revealed a positive enrichment of cell cycle promoting gene signatures in siCtrl samples predominantly when compared to siNFATc1+TGFβ conditions (Fig. S2A), suggesting that combined TGFβ treatment and NFATc1 depletion has synergistic impact on reduction of tumor cell proliferation in PDAC cells.

In accordance with the enrichment of an apoptosis-related gene signature in siNFATc1+TGFβ compared to siCtrl+TGFβ samples (Fig. 2a) and elevated cell counts in sub-G1 phase upon combined NFATc1 knockdown and TGFβ treatment (Fig. 2b), NFATc1 knockdown in the presence of TGFβ resulted in maximal expression of cleaved caspases 3, 7 and poly ADP-ribose polymerase (Figs. 2d and S2B). Consistently, Annexin/PI staining and subsequent flow cytometry in NKC cells showed three-fold increased apoptosis induction upon NFATc1 depletion, but the pro-apoptotic effects of NFATc1 knockdown could be further enhanced by TGFβ treatment (Figs. 2e and S2C), suggesting that NFATc1 activity interferes with TGFβ-dependent regulation of pro-apoptotic gene signatures.

Together, these data not only confirm the positive role of NFATc1 in the regulation of tumor cell proliferation^18,55,56^ but also suggest that NFATc1 at least partially interferes with the tumor growth-inhibiting function of TGFβ.

### NFATc1-dependent transcription programs are linked to bad outcome of PDAC patients

**Fig. 3:**
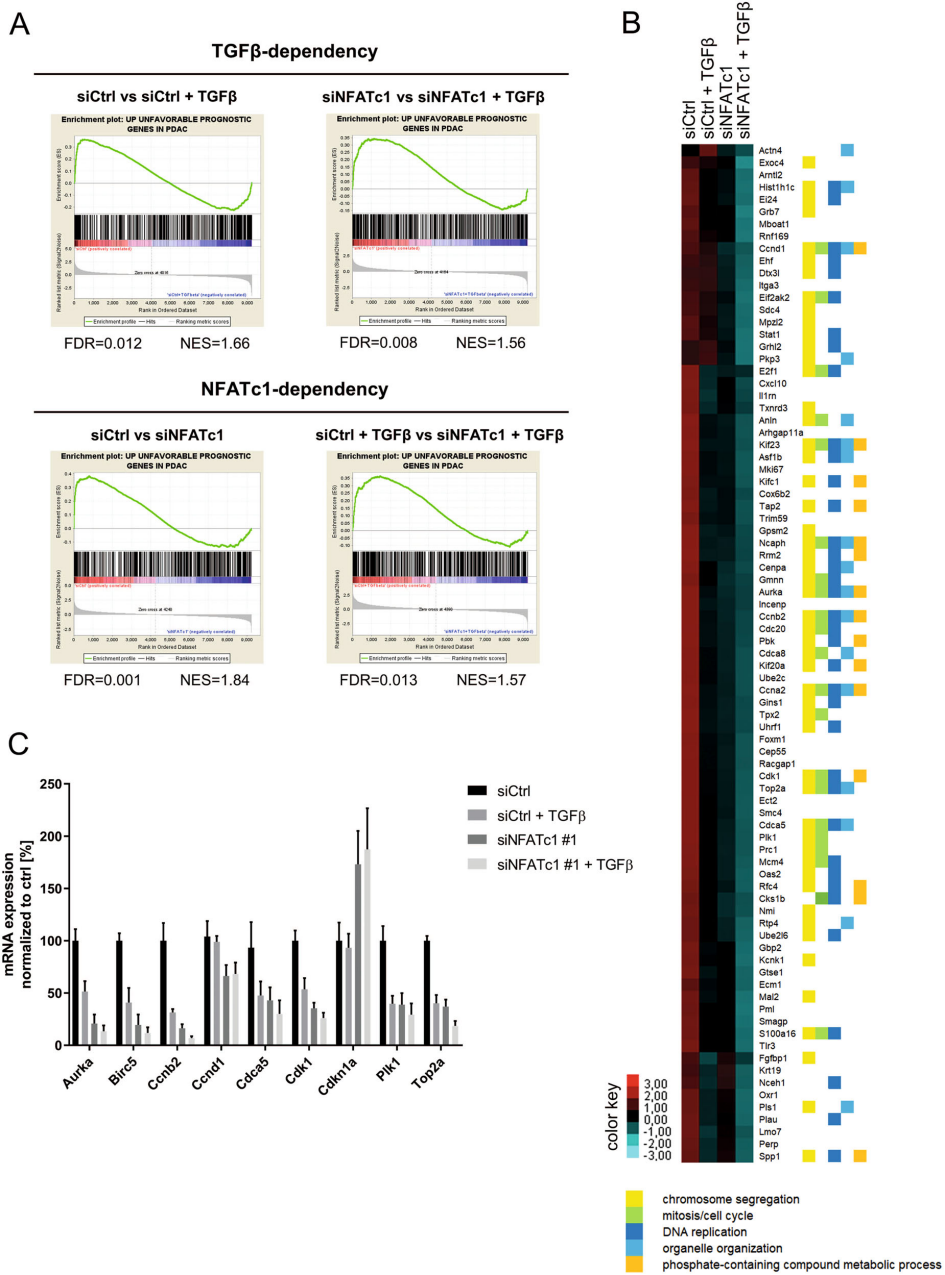
NFATc1-dependent transcription programs are linked to bad outcome of pancreatic ductal adenocarcinoma (PDAC) patients. **a** Alignment of unfavorable prognostic genes in PDAC dataset with RNA-seq data (Fragments Per Kilobase Million >5) by gene set enrichment analysis demonstrates loss of tumor features upon NFATc1 knockdown and transforming growth factor β (TGFβ). **b** Heatmap displaying *z* scores of leading edge regulated unfavorable genes. Gene ontology analysis confirmed their functional involvement in proliferation. **c** The expression of a selection of these genes was tested via quantitative real-time polymerase chain reaction in NKC-II cells in the presence and absence of TGFβ and NFATc1, respectively (*n* = 3)

Based on the significant tumor-promoting role of NFATc1-dependent transcription in PDAC, we proposed that NFATc1 activity may be associated with a bad prognosis of PDAC patients. To verify this hypothesis, we employed publically available data provided by the human protein atlas and TCGA^45,46^ and compared our RNA-seq results to genes associated with an unfavorable PDAC prognosis. Among the 669 annotated genes associated with unfavorable prognosis, 427 genes (63.8%) passed the FPKM threshold (FPKM > 5) and were included for further analysis. Importantly, the vast majority of genes were found to be downregulated upon sole TGFβ treatment or NFATc1 knockdown, while the lowest *z* scores of genes were detected in the siNFATc1+TGFβ group (Figs. 3a and S3A). Further GO analysis of the regulated leading edge unfavorable prognostic genes revealed a critical involvement in functional processes associated with proliferation (e.g., chromosome segregation, mitosis, cell cycle, and DNA replication) for 67.5% (56/83) of all hits (Fig. 3b). Consequently, we selected differentially expressed target genes from our RNA-seq study that were linked to bad PDAC outcome as defined by TCGA (patient groups *n* = 87) (Figs. 3b and S4) and verified their expression in an independent experimental setting. While we observed a strong reduction of mRNA expression upon either TGFβ treatment or NFATc1 knockdown, the lowest expression of pro-proliferative target genes was detected upon combined NFATc1 depletion and TGFβ treatment (Figs. 3c and S3B, C), suggesting synergistic or additive effects of both components in the regulation of proliferative transcriptional programs. In contrast, *Cdkn1a* (encoding for the negative cell cycle regulator p21), which was not associated with a poor PDAC prognosis (Fig. S4), was unaffected upon TGFβ treatment but was found to be highly induced upon NFATc1 knockdown both at the mRNA and protein levels (Figs. 2d, 3c, S2B and S3C), suggesting an involvement of NFATc1 in *Cdkn1a* repression.

Consistent with our findings depicted in Fig. 2, these data imply that NFATc1 controls transcription of pro-proliferative target genes that are strongly linked to a poor prognosis of PDAC patients and demonstrate that NFATc1 compromises the execution of predominantly tumor-suppressive TGFβ-dependent transcription programs in PDAC cells.

### NFATc1- and TGFβ-mediated target gene regulation occurs at the level of chromatin

**Fig. 4:**
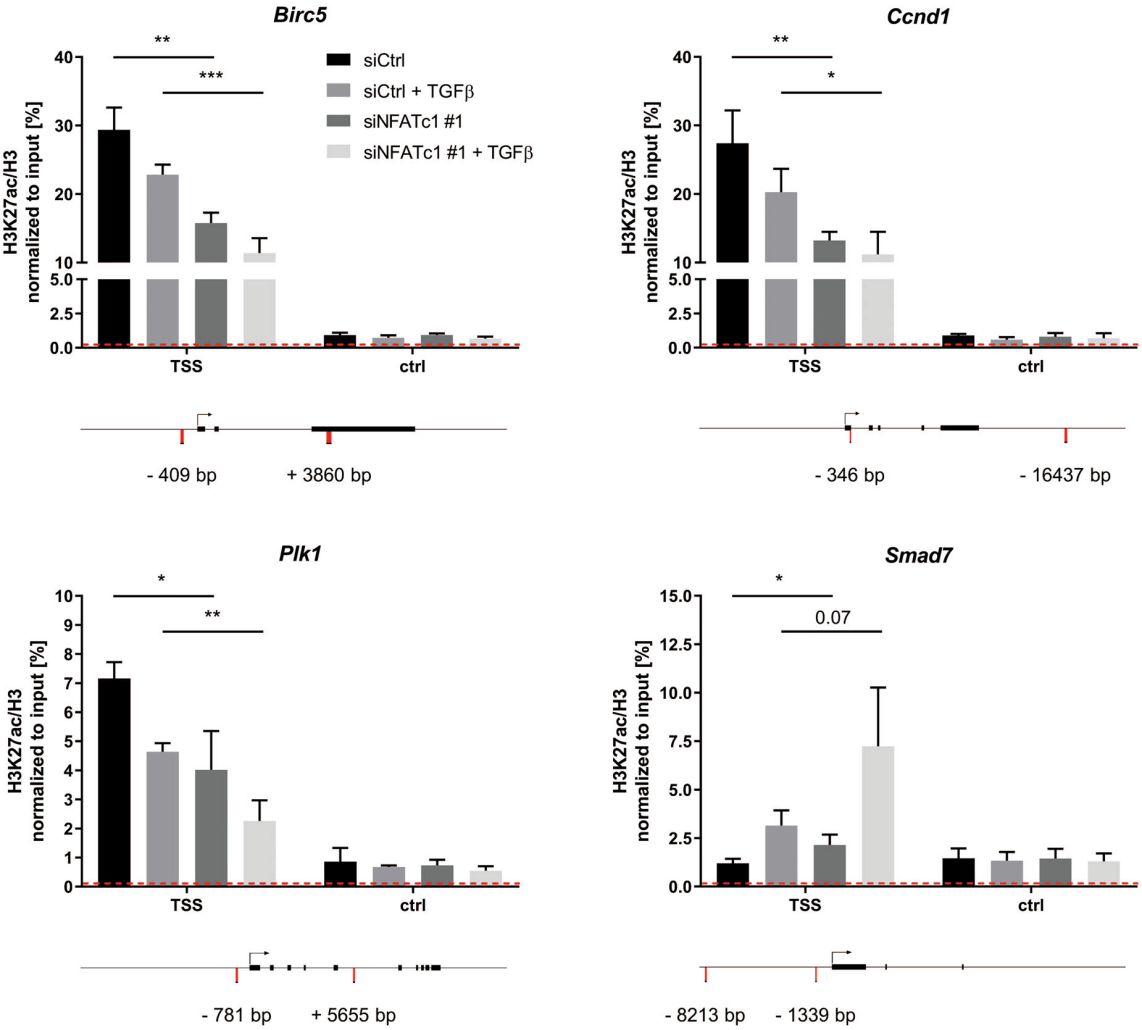
NFATc1- and transforming growth factor β (TGFβ)-mediated target gene regulation occurs at the level of chromatin. Chromatin immunoprecipitation experiments (*n* = 3) demonstrating H3K27ac/H3 occupancy at transcriptional start sites (TSS) and control region (promoter/intragenic) of a subset of target genes identified in Fig. 3 and implicated in proliferation and growth. H3K27ac occupancy at the *Smad7* gene was used as control. Dotted line represents IgG. Schemes display location of respective primers in relation to TSS

Next we aimed to elucidate whether TGFβ- and/or NFATc1-dependent transcriptional regulation was accompanied by local alterations of the chromatin landscape. To this end, we performed ChIP analyses in NKC cells and investigated the occupancy of H3K27ac (a histone mark indicative of transcriptional activation) at the transcriptional start sites (TSS) of a subset of jointly TGFβ/NFATc1-regulated unfavorable prognosis target genes. While global H3K27ac levels remained unchanged upon TGFβ treatment and NFATc1 depletion (Fig. S5A), we observed differential H3K27ac occupancy specifically at the TSS of *Birc5*, *Ccnd1*, and *Plk1* (Figs. 4 and S5B, C). PDAC cell stimulation with TGFβ or NFATc1 depletion each reduced H3K27ac occupancy at the TSS. However, and in line with our expression analyses (Figs. 3c and S3C), the strongest reduction of H3K27ac occupancy at the TSS was observed upon TGFβ treatment in the context of NFATc1 knockdown, indicating that NFATc1 antagonizes TGFβ-driven local chromatin modification and remodeling processes. Importantly, ChIP analysis at the TSS region of *Smad7* revealed inverse H3K27ac regulation with increased H3K27ac occupancy upon TGFβ treatment or NFATc1 depletion alone but in particular upon combined NFATc1 loss and TGFβ stimulation (Figs. 4 and S5B, C), further suggesting that NFATc1 blocks TGFβ-induced target gene activation. Together, these data indicate that NFATc1- and TGFβ-dependent transcriptional control of unfavorable prognostic genes in PDAC is indeed mediated at the level of chromatin regulation.

### NFATc1 activity promotes PanIN progression

**Fig. 5:**
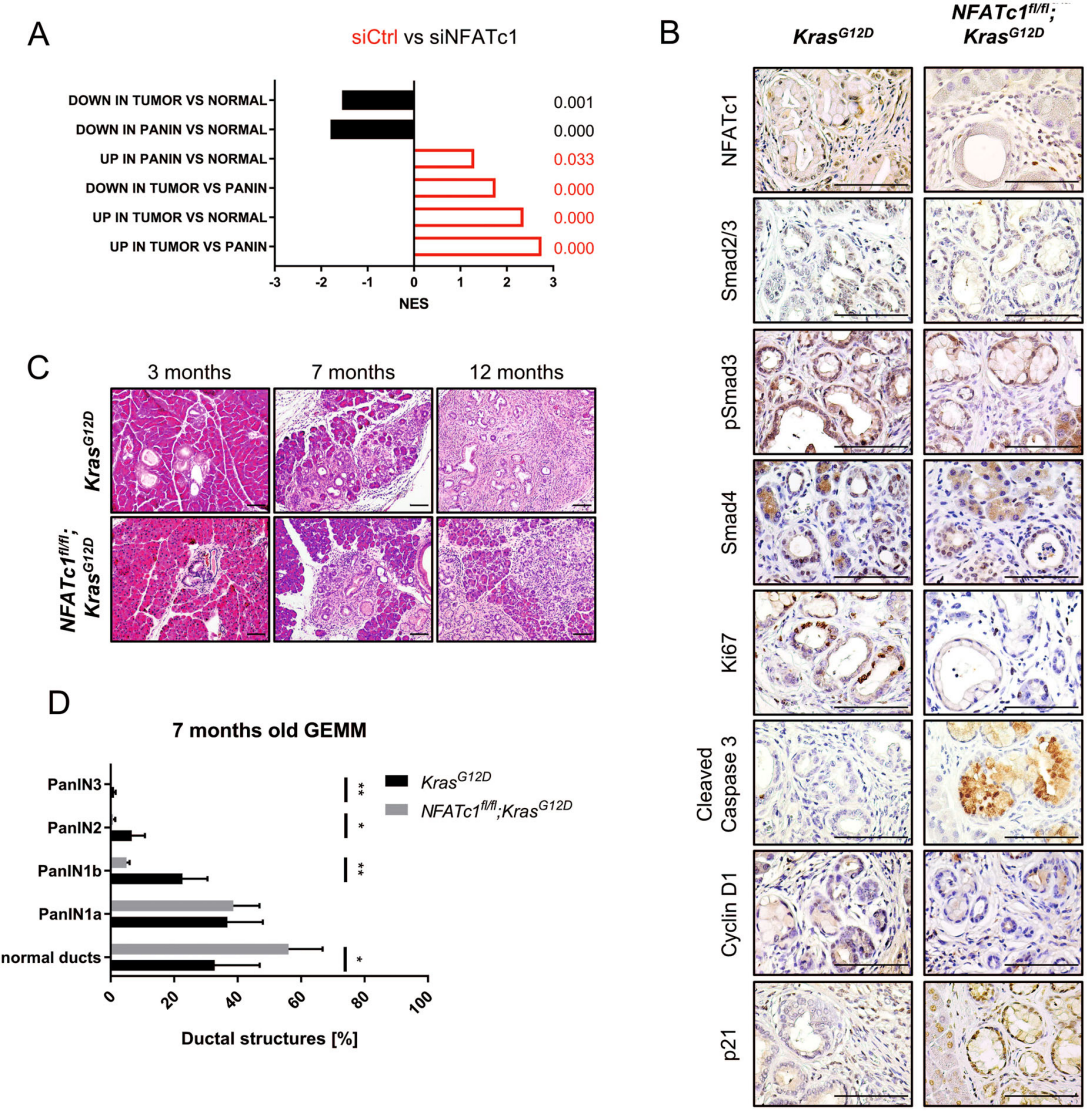
NFATc1 activity promotes pancreatic intraepithelial (PanIN) progression. **a** Gene set enrichment analysis matches RNA-seq data (Fragments Per Kilobase Million >5) to gene signatures associated with tumor (pancreatic ductal adenocarcinoma), PanIN stage, or normal, healthy tissue. Log2fold change of ≥0.5; ≤−0.5 was set to differentiate the respective groups. **b** Immunohistochemical stainings (×400, scale bar: 100 µm) in preneoplastic lesions of 7-month-old *Kras*^*G12D*^ and *NFATc1*^*fl/fl*^*;Kras*^*G12D*^ mice. **c** Representatives hematoxylin and eosin stainings (×100, scale bar: 100 µm) of 3-month-, 7-month-, and 12-month-old genetically engineered mouse model (GEMM). **d** Quantification of ductal structures in GEMM (7 months; *n* = 5 per genotype)

Based on the NFATc1-dependent promotion of transcription programs associated with an unfavorable PDAC prognosis, we next sought to dissect whether NFATc1-regulated gene signatures can be linked to certain stages of PDAC evolution. To this end, we compared our RNA-seq data to gene signatures associated with either healthy or diseased (tumor or PanIN lesions) pancreatic tissue^53^. While signatures characterizing healthy pancreatic tissue were enriched upon NFATc1 depletion, signatures characterizing PDAC and its precursor lesions were significantly enriched in control samples (Fig. 5a). To validate the underlying biological significance, we intercrossed GEMMs with constitutive activation of Kras (*Kras*^*G12D*^ mice)^28^ with mice harboring pancreas-specific NFATc1 deficiency (*NFATc1*^*fl/fl*^ mice) to generate *NFATc1*^*fl/fl*^*;Kras*^*G12D*^ mice^24,25^. IHC revealed absence of NFATc1 staining in the epithelial compartment of *NFATc1*^*fl/fl*^*;Kras*^*G12D*^ animals, while infiltrating immune cells retained NFATc1 expression (Fig. 5b). In accordance with previous studies of our group characterizing the impact of NFATc1 specifically in the context of inflammation-associated PDAC initiation and progression^24,25^, histological comparison of pancreata derived from 3-, 7-, and 12-month-old *Kras*^*G12D*^ and *NFATc1*^*fl/fl*^*;Kras*^*G12D*^ mice showed a strong decrease of premalignant acinar-to-ductal metaplasia formation and advanced PanIN lesions in NFATc1-deficient animals (Fig. 5c, d). In line with the enrichment of gene signatures of the healthy pancreas in NFATc1-depleted NKC-II cells (Fig. 5a), *NFATc1* knockout in our GEMM sufficiently hampered pancreatic carcinogenesis, even in the absence of inflammatory insults. Importantly, TGFβ signaling in PanIN lesions was active in both models, as indicated by the nuclear expression of Smad proteins (Fig. 5b). However, we observed reduced Ki67 and Cyclin D1 staining and enhanced Cleaved Caspase 3 and p21 expression in PanIN lesions of *NFATc1*^*fl/fl*^*;Kras*^*G12D*^ compared to *Kras*^*G12D*^ mice, suggesting decreased proliferation and enhanced apoptotic activity in NFATc1-deficient animals (Fig. 5b).

Taken together, studies conducted in NFATc1 wild type expressing *Kras*^*G12D*^ and in *NFATc1*^*fl/fl*^*;Kras*^*G12D*^ mice faithfully recapitulate features of NFATc1-dependent signatures identified in primary PDAC cells and suggests that NFATc1 loss in epithelial pancreatic cells delays PanIN progression.

## Discussion

Herein we examined the interplay between TGFβ signaling and NFATc1 activity in the regulation of transcriptional programs associated with PDAC progression. Based on previous studies characterizing NFATc1 as a critical mediator of the TGFβ switch from a suppressor to a promoter of PDAC progression^18^, we expected that TGFβ treatment in the presence of NFATc1 augments the transcription of pro-tumorigenic gene programs in PDAC cells. Surprisingly, our transcriptomic data and functional analysis link TGFβ signaling with downregulation of proliferative gene signatures and reduced PDAC cell proliferation, even in the presence of oncogenic NFATc1. Although unlimited tumor growth represents only one of the several tumor-promoting hallmarks^57^ and TGFβ treatment might positively regulate other oncogenic features in these cells (e.g., migration), the reduced expression of genes related to an unfavorable prognosis of PDAC patients in the presence of the cytokine suggests that active TGFβ signaling in primary tumor cells derived from *NKC p48* mice retains tumor-suppressive functions. This obvious discrepancy to previous studies can at least partially be assigned to the constitutive activation of NFATc1 in our cell system. While TGFβ treatment in cells harboring endogenous NFAT expression results in nuclear accumulation of the TF and subsequent induction of oncogenic transcription programs^18^, primary PDAC cells from *NKC p48* mice harbor constitutive nuclear NFATc1, even in a treatment-naive state^24^. Consequently, in this setting, TGFβ treatment does not further boost NFATc1 expression or activity but might signal via NFAT-independent canonical pathways with different transcriptional and functional readouts. Indeed, the tumor-suppressor gene *Smad4*, whose inactivation is widely accepted as an important genetic hit determining the TGFβ switch, is undisturbed in our cell system. Moreover, and in contrast to PDAC cells utilized in our previous study^18^, *NKC* cells harbor wild-type expression of p53. Mutation of this master tumor-suppressor gene has been described to synergize with Ras activation in inducing the TGFβ switch by driving non-canonical/Smad-independent TGFβ signaling^58,59^. Hence, our findings underscore the strong context-dependency of TGFβ-mediated transcription and function in PDAC progression.

Similar to TGFβ treatment alone, and in line with previous reports highlighting the strong oncogenic potential of NFATc1^18,24–26,60^, TF depletion was associated with downregulation of unfavorable prognostic genes, enhanced expression of gene sets linked to less advanced disease stages, and delayed pancreatic carcinogenesis in vivo. Importantly, the pro-tumorigenic consequences of NFATc1 activity in PDAC were not only limited to activation of transcriptional programs involved in positive regulation of tumor growth but also comprise the blockade of apoptosis-associated gene signatures. NFAT-dependent repression of tumor failsafe programs in PDAC has been previously reported in the context of transcriptional inactivation of the tumor-suppressor gene *Cdkn2b*, which negatively controls cell cycle progression and induces cellular senescence^30^. In this report, we demonstrated NFAT-dependent recruitment of the histone methyltransferase Suv39H1 to the *Cdkn2b* promoter, thus resulting in H3K9me3 and heterochromatin protein 1γ (HP1γ)-mediated local heterochromatin formation and gene silencing^30^. Although it remains elusive whether the herein observed NFAT-mediated regulation of apoptosis-related gene signatures occurs directly (via DNA binding and, e.g., recruitment of repressive chromatin regulators) or indirectly (via intermediate players), these findings emphasize that NFAT TFs are not only capable of inducing pro-tumorigenic transcription programs in PDAC but also repress cellular mechanisms that constrain unlimited tumor growth.

On top of the individual anti-tumorigenic consequences of TGFβ treatment or NFATc1 loss, combined NFATc1 knockdown and TGFβ treatment in our PDAC cells displayed maximal reduction or induction of proliferation and apoptosis features, respectively. Importantly, this synergism was particularly evident for genes related to an unfavorable prognosis of PDAC patients. Hence, we postulate that TGFβ competes with NFATc1 for the regulation of tumor-promoting target genes, for instance, by recruiting chromatin regulatory proteins with opposing consequences on histone acetylation, chromatin accessibility, and subsequent transcriptional activity. Histone acetylation constitutes a highly dynamic and reversible process, which is fine-tuned by chromatin-modifying proteins that compete for lysine residues to either add or remove acetyl groups on histones^61,62^. While histone acetylation, which diminishes chromatin compaction, is associated with transcriptional activity, deacetylation blocks transcription^62,63^. In accordance with our expression data, chromatin studies conducted at unfavorable prognostic target genes revealed the lowest H3K27ac occupancy upon simultaneous NFATc1 depletion and TGFβ treatment, indicating that NFATc1- and TGFβ-dependent transcriptional control of these genes indeed occurs at the chromatin level. Importantly, reduced H3K27ac occupancy upon simultaneous TGFβ treatment and NFATc1 depletion can either be the consequence of reduced histone acetylation (installed by histone acetyltransferases (HATs), e.g., p300, CBP) or a result of increased histone deacetylation (mediated by histone deacetylases (HDACs))^62^. HATs and HDAC proteins are critically involved in PDAC evolution and therapeutic strategies targeting misdirected histone acetylation are under preclinical and clinical evaluation^64^. Importantly, the recruitment of chromatin modifiers for subsequent changes in histone modification at specific target genes needs to occur in a temporally and spatially restricted manner and is controlled by hierarchical signaling events that convert extracellular or intracellular stimuli into a transcriptional program^65^. Consistently, we have previously shown that NFATc1 is capable of recruiting the HAT p300 for subsequent histone acetylation on tumor-promoting target genes^18^. Notably, activation of the TGFβ/SMAD pathway installs or removes histone acetylation via recruitment of HATs or HDACs for subsequent transcriptional activation or repression, respectively, in a highly context-dependent manner^66,67^. Emphasizing the tumor biological impact of TGFβ-dependent control of chromatin regulatory proteins in PDAC, we recently showed that HDAC inhibition abrogates TGFβ-induced EMT transcriptional programs^68^. Given the critical involvement of HATs and HDACs in TGFβ- and NFATc1-controlled transcription and based on our findings of reduced H3K27ac occupancy on jointly regulated target genes, we propose that simultaneous induction of TGFβ-dependent and HDAC-mediated H3K27 deacetylation synergizes with reduced de novo H3K27 acetylation upon NFATc1 depletion, thus resulting in decreased transcription of unfavorable prognostic genes in *NKC* cells. Although the exact mechanisms by which TGFβ and NFATc1 elicit opposing effects on pro-tumorigenic transcription programs remain to be elucidated, our findings underscore the strong context-dependency of TGFβ- and NFATc1-controlled transcription programs and emphasize that pharmacological targeting of these programs in PDAC requires careful consideration of the molecular makeup of a given tumor cell.

## Supplementary information

Supplementary Information

Supplementary Figure 1

Supplementary Figure 2

Supplementary Figure 3

Supplementary Figure 4

Supplementary Figure 5
