# Supplementary material for: Aberrant NFATc1 signaling counteracts TGFβ-mediated growth arrest and apoptosis induction in pancreatic cancer progression
Source: Cell Death Dis. 2019 Jun 6;10(6):446. doi: 10.1038/s41419-019-1682-2 (PMC6554303; doi:10.1038/s41419-019-1682-2)
Supplement: Supplementary file 1 — Supplementary Information [file 41419_2019_1682_MOESM1_ESM.docx]

**Supplementary files**

**Supplementary figures**

**Supplementary Figure S1: a)** Experimental illustration of treatment conditions. **b)** QRT-PCR conducted in the replicates used for RNA-seq. **c)** Western blot treatment control for RNA-seq. **d)** PCA plot and sample-to-sample distances of four distinct conditions. **e)** Number of differentially up- and downregulated genes (log2foldchange ≥ 1; ≤ -1; q ≤ 0.05; FPKM > 5).

**Supplementary Figure S2:** **a)** GSEA of cell cycle-related gene sets. **b)** Western blot demonstrating expression of cell cycle arrest- and apoptosis associated markers in NKC-II cells upon NFATc1 knockdown using an alternative siRNA sequence and verification in NKC-VI and NKC-VIII. **b)** Apoptosis induction was investigated via Annexin/PI staining followed by flow cytometry (n=3) in NKC-VI and NKC-VIII cells.

**Supplementary Figure S3:** **a)** Z-score heatmap of unfavorable prognostic genes (log2fold changes ≥ 1; ≤ -1; q ≥ 0.05; FPKM > 5). **b)** QRT-PCR to demonstrate treatment and knockdown control (corresponding to Figure 3C) (n=3). **c)** Gene expressions demonstrated in Figure 3C were validated with an alternative siRNA (n=3).

**Supplementary Figure S4:** TCGA survival data for NFATc1 targets implicated in unfavorable prognosis for PDAC patients significantly correlate with reduced survival, except for CDKN1A (patient cohort n=87, respectively).

**Supplementary Figure S5:** **a)** Western Blot displaying constant global H3K27ac level upon TGFβ treatment and NFATc1 depletion in NKC-II cells. **b)** Western Blot treatment controls for ChIP experiment depicted in Figure 4. **c)** QRT-PCRs of ChIP experiments in NKC-VI and NKC-VIII cells. Dotted lines indicate average IgG levels.

**Supplementary tables**

**Supplementary table 1: QRT-PCR primer sequences for cDNA.**

| **Target** | **Direction** | **Sequence** |
| --- | --- | --- |
| Aurka | forward | GCCCCTTGGAACAGTCTATAG |
|  | reverse | CTCTGGCTTAATGTCTCTGTGG |
| Birc5 | forward | TCGCCACCTTCAAGAACTG |
|  | reverse | CCCAGCCTTCCAATTCCTTA |
| Ccnb2 | forward | CCTCAGAACACCAAAGTACCAG |
|  | reverse | CCTTCATGGAGACATCCTCAG |
| Ccnd1 | forward | CCATGACTCCCCACGATTTC |
|  | reverse | GGTGGGTTGGAAATGAACTTC |
| Cdca5 | forward | CTCTGGCTTAATGTCTCTGTGG |
|  | reverse | TTCTTTCGTGGGTACCTTGAG |
| Cdk1 | forward | AAGTGTGGCCAGAAGTCGAG |
|  | reverse | TCGTCCAGGTTCTTGACGTG |
| Cdkn1a | forward | CAGATCCACAGCGATATCCAG |
|  | reverse | AGAGACAACGGCACACTTTG |
| NFATc1 | forward | GCCTTTTGCGAGCAGTATCT |
|  | reverse | GCTGCCTTCCGTCTCATAGT |
| Plk1 | forward | CACGTCGTAGGCTTCCATGA |
|  | reverse | GAATGACCTGATTGCGGTGC |
| Rplp0 | forward | TGGGCAAGAACACCATGATG |
|  | reverse | AGTTTCTCCAGAGCTGGGTTGT |
| Smad7 | forward | TGCTCCCATCCTGTGTGTTAAG |
|  | reverse | TCAGCCTAGGATGGTACCTTGG |
| Top2a | forward | TGACAAGCGAGAAGTGAAGG |
|  | reverse | GCTACCCACAAAATTCTGCG |

**Supplementary table 2: Antibodies used for IHC.**

| **Target** | **Company** | **No** | **Dilution** |
| --- | --- | --- | --- |
| Cyclin D1 | BD Biosciences | 554180 | 1:200 |
| Cleaved Caspase 3 | CST | 9661 | 1:200 |
| HA-Tag | CST | 3724 | 1:200 |
| Ki67 | NeoMarkers | RM-9106-S0 | 1:600 |
| NFATc1 | abcam | ab2796 | 1:100 |
| p21 | abcam | ab2961 | 1:300 |
| Smad2/3 | BD Biosciences | 610842 | 1:200 |
| pSmad3 | abcam | ab51177 | 1:50 |
| Smad4 | CST | 46535 | 1:50 |
| TGFβ | abcam | ab66043 | 1:100 |

**Supplementary table 3: Antibodies used for western blot.**

| **Target** | **Company** | **No** | **Dilution** |
| --- | --- | --- | --- |
| Caspase 7 | CST | 9492 | 1:1000 |
| Cleaved Caspase 3 | CST | 9661 | 1:1000 |
| H3K27ac | Genetex | GTX128944 | 1:1000 |
| HA-Tag | CST | 2367 | 1:1000 |
| NFATc1 | Santa Cruz | sc-7294 | 1:500 |
| p21 | Santa Cruz | sc-6246 | 1:500 |
| Parp | CST | 9542 | 1:1000 |
| Smad4 | Santa Cruz | sc-7966 | 1:500 |
| Smad2/3 | BD Biosciences | 610842 | 1:1000 |
| pSmad2/3 | CST | 8828 | 1:1000 |
| Snai1 | CST | 3895 | 1:1000 |
| β-Actin | Sigma | A3854 | 1:40000 |

**Supplementary table 4: QRT-PCR primer sequences for ChIP experiments.**

| **Target** | **Direction** | **Sequence** |
| --- | --- | --- |
| Birc5_TSS | forward | TGCTTCCCTTTGGTCTGAGC |
|  | reverse | ATGGAGGCGGGGTCTGTAT |
| Birc5_intragenic | forward | CAGCCAGGCATGAAGAGTCA |
|  | reverse | AGGCTGCCTGCTTAGAGTTG |
| Ccnd1_TSS | forward | TCCAGGTGGCCACGATTTTC |
|  | reverse | GCGCCCTCCGTATCTTACTT |
| Ccnd1_Promoter | forward | TCCAGCCCAGTTTCTTGCTC |
|  | reverse | CTGAGTGCTCCCCTACCTCT |
| Plk1_TSS | forward | CCACACAACTTGGAACAACCC |
|  | reverse | CTGCCGGTCAAACAAGACCT |
| Plk1_intragenic | forward | ACAGACTCAGGTTTGAAATCCCC |
|  | reverse | GATGATAAATGCAGTGGCACAGTC |
| Smad7_TSS | forward | AGGGGAATGGTCAGAGAACCTA |
|  | reverse | AGGGCTGGGGGTATAATTCCTT |
| Smad7_Promoter | forward | TTGGTCATTCTGAAATAGTGCCTC |
|  | reverse | AAATGCTGGCAACCCAATGAG |
