# Supplementary figures and images for: Aberrant NFATc1 signaling counteracts TGFβ-mediated growth arrest and apoptosis induction in pancreatic cancer progression
Source: Cell Death Dis. 2019 Jun 6;10(6):446. doi: 10.1038/s41419-019-1682-2 (PMC6554303; doi:10.1038/s41419-019-1682-2)

A

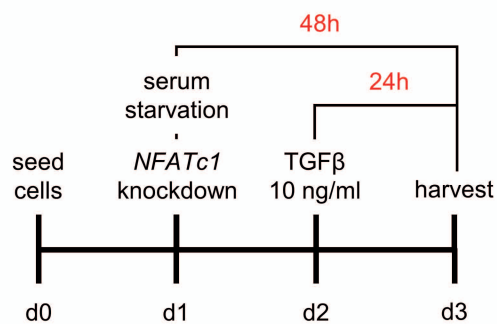

B

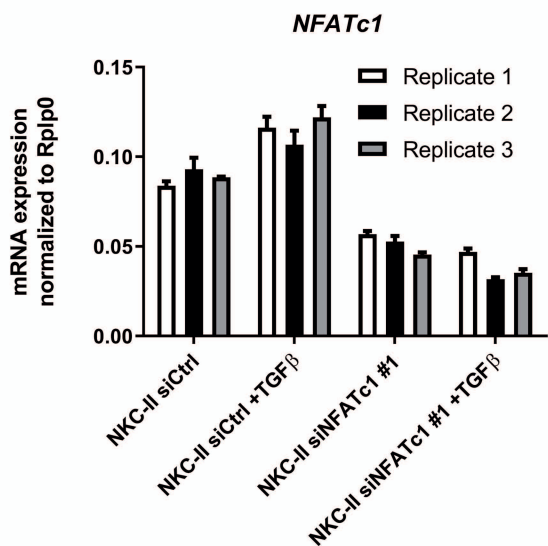

C

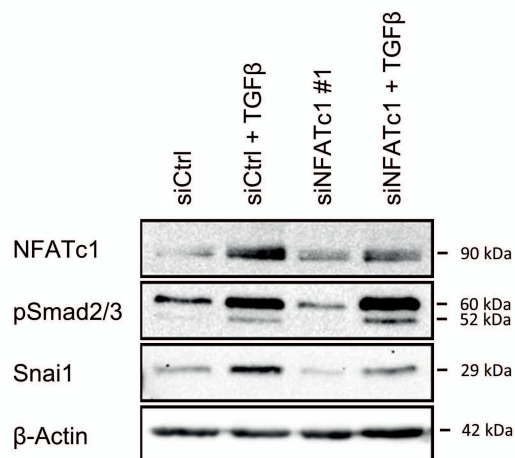

D

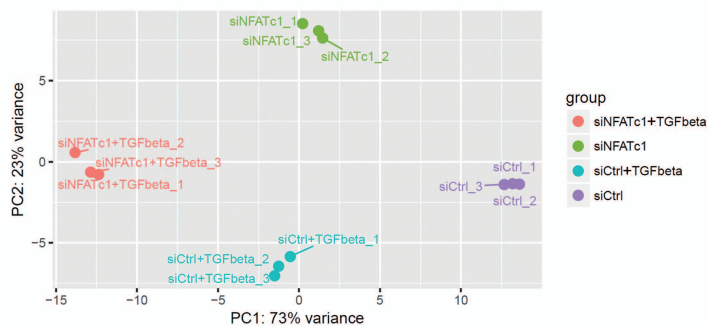

E

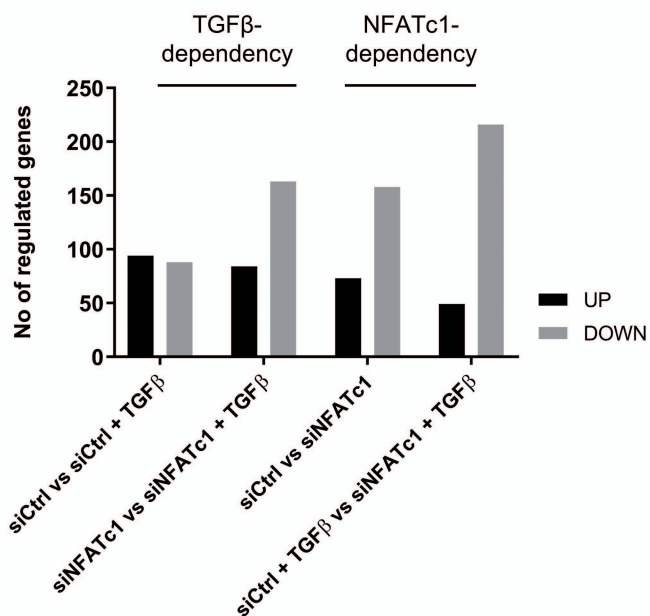

Supplementary Figure 1

Supplement: Supplementary file 2 — Supplementary Figure 1 [file 41419_2019_1682_MOESM2_ESM.pdf]

siCtrl vs siNFATc1 + TGFβ

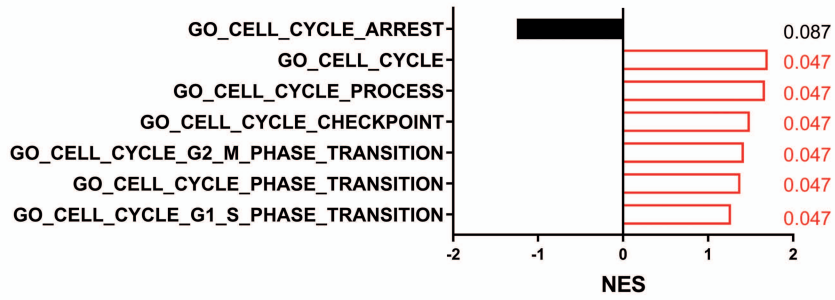

# B

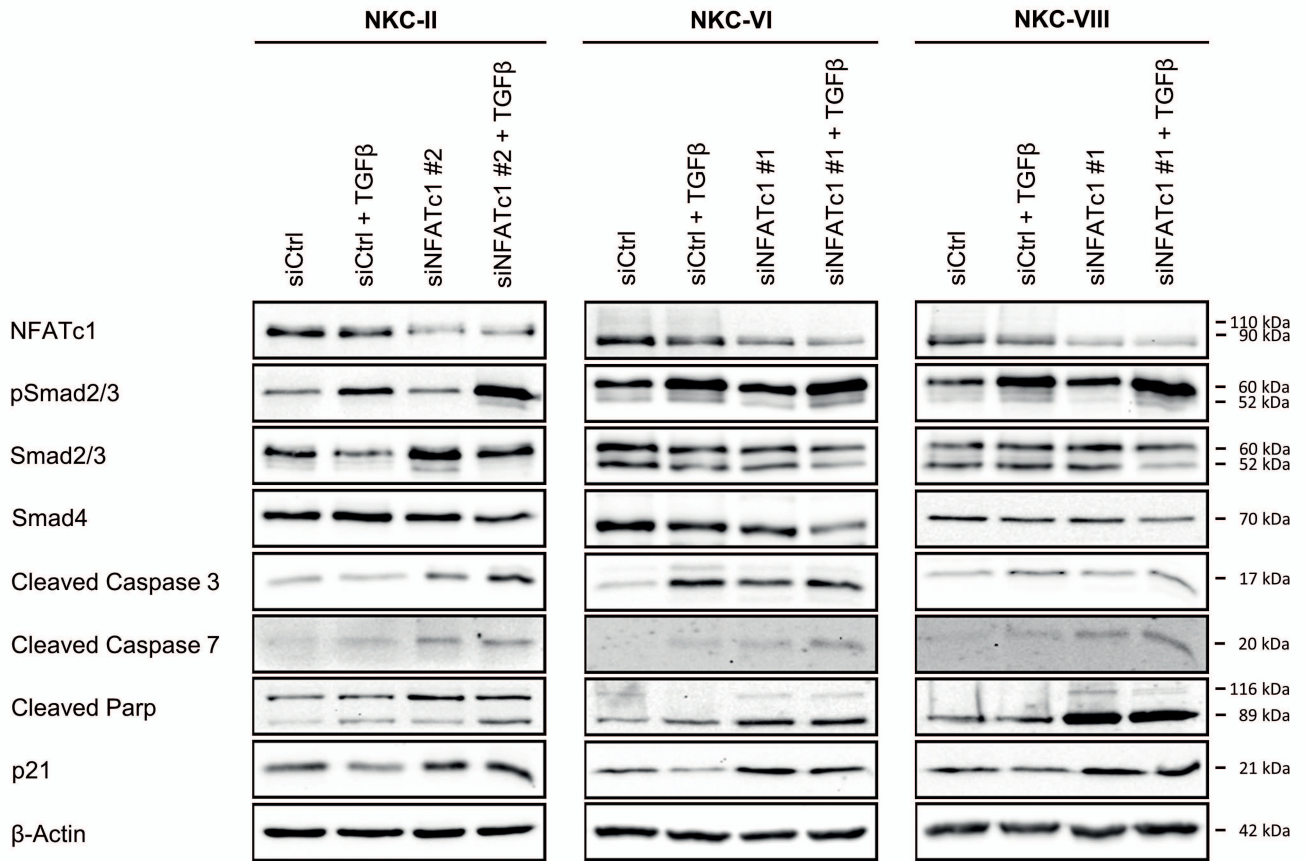

C

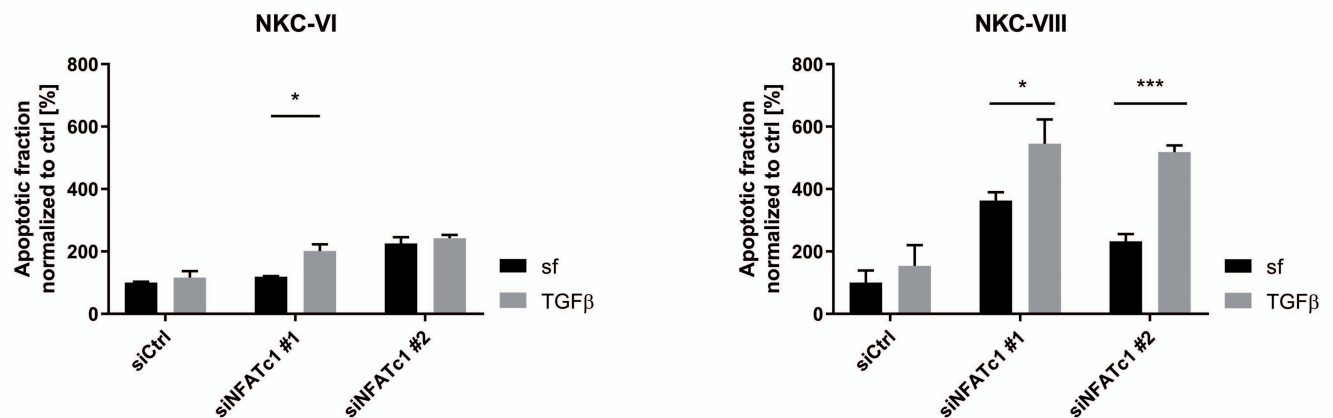

## Supplementary Figure 2

Supplement: Supplementary file 3 — Supplementary Figure 2 [file 41419_2019_1682_MOESM3_ESM.pdf]

A

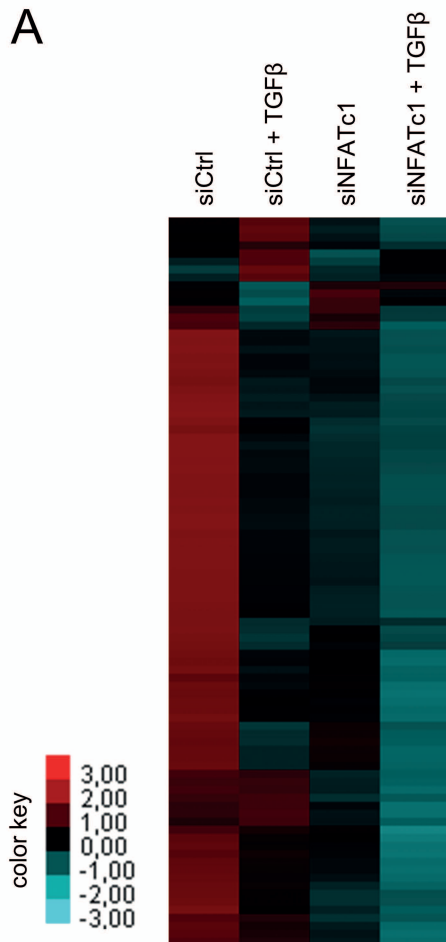

B

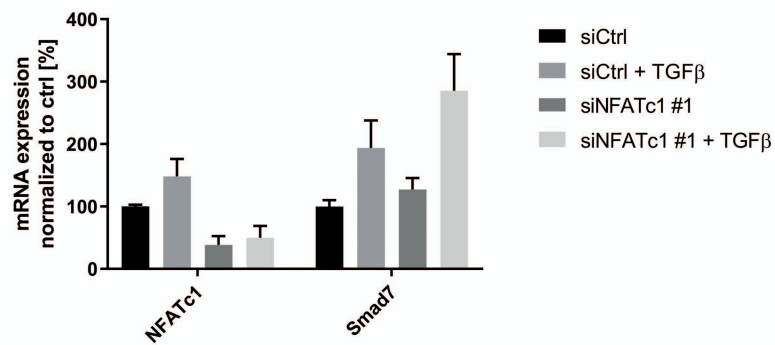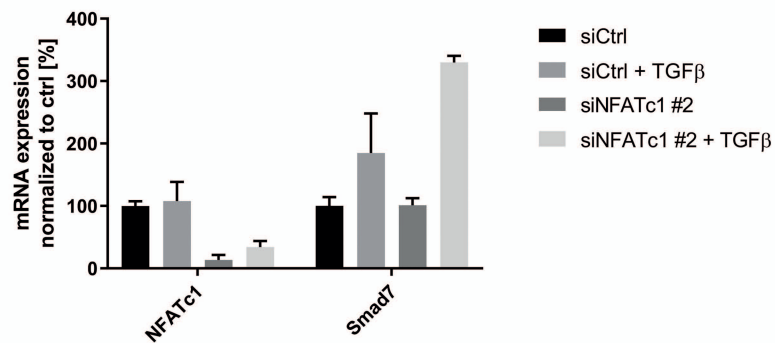

C

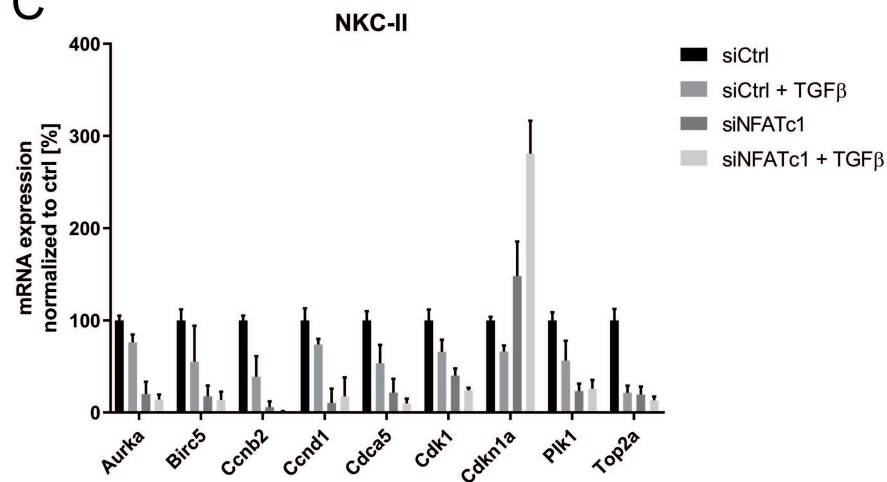

Supplementary Figure 3

Supplement: Supplementary file 4 — Supplementary Figure 3 [file 41419_2019_1682_MOESM4_ESM.pdf]

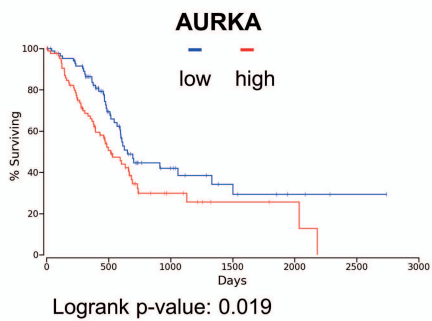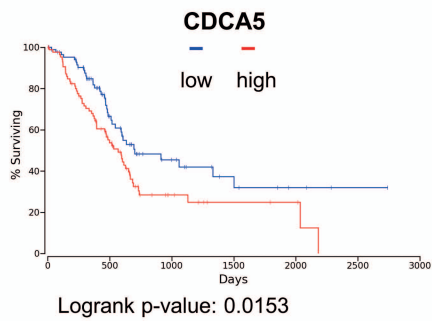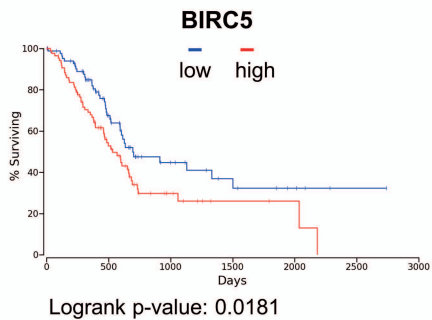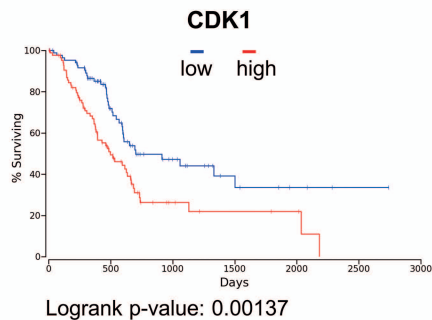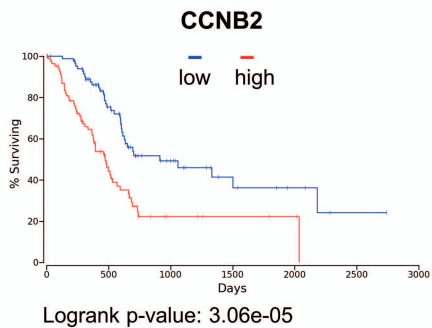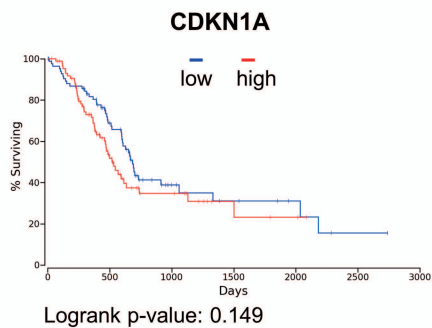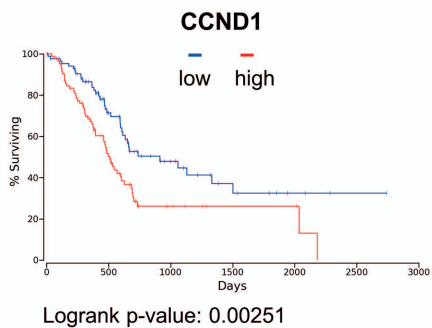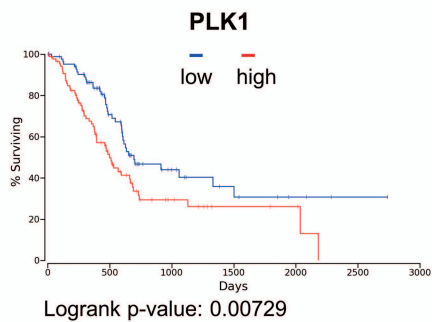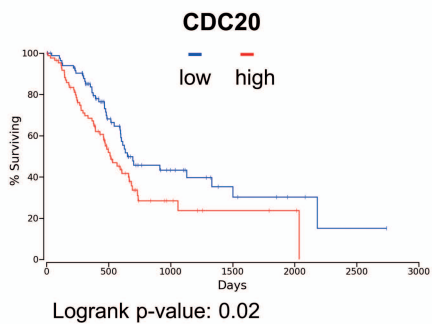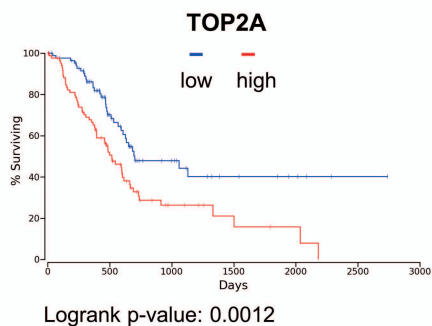

**Supplementary Figure 4**

Supplement: Supplementary file 5 — Supplementary Figure 4 [file 41419_2019_1682_MOESM5_ESM.pdf]

A

NKC-II

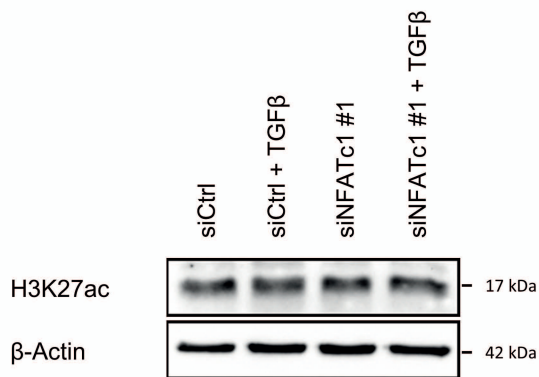

B

NKC-II

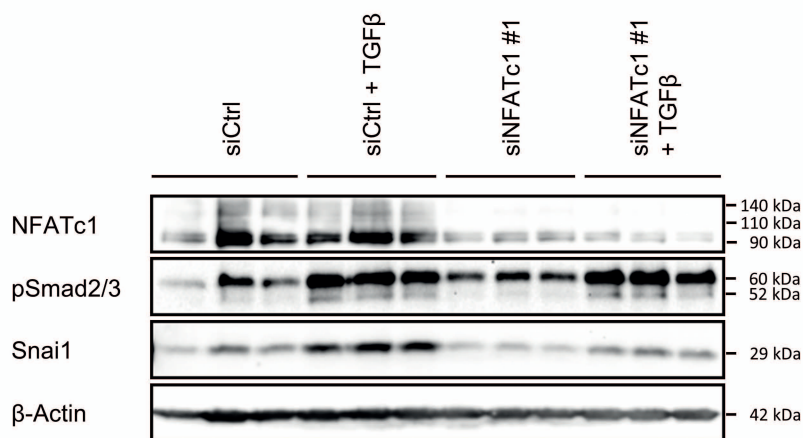

C

NKC-VI

*Birc5*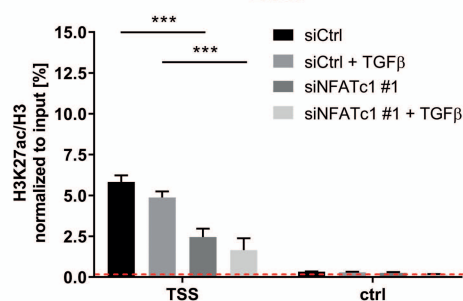*Ccnd1*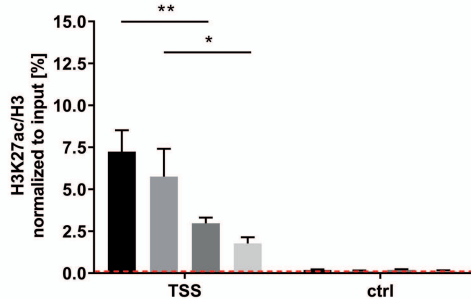*Plk1*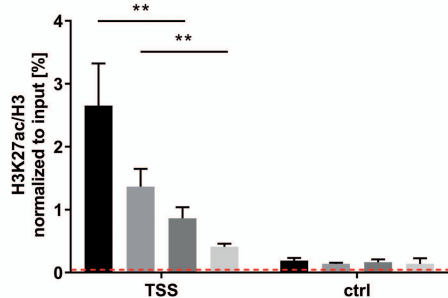*Smad7*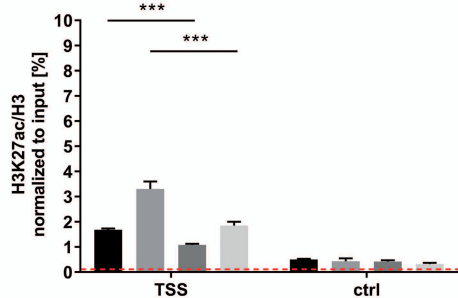

NKC-VIII

*Birc5*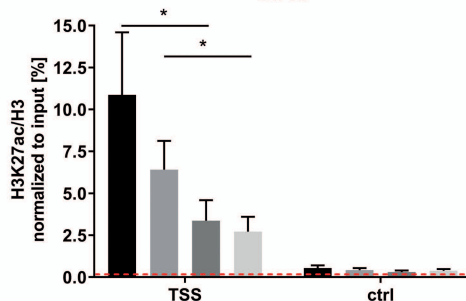*Ccnd1*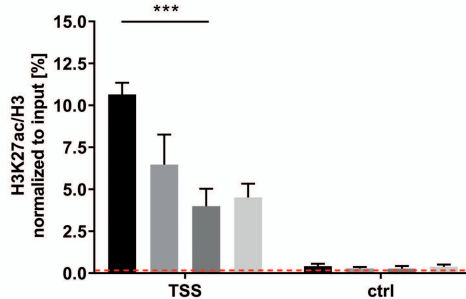*Plk1*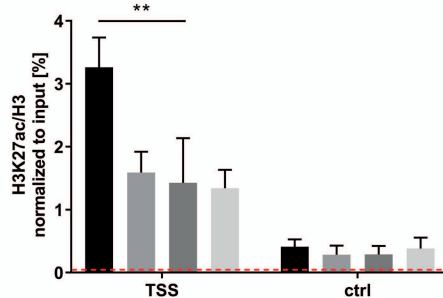*Smad7*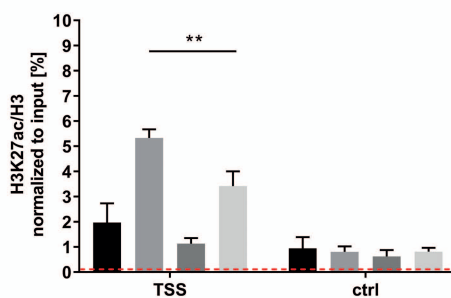

Supplementary Figure 5

Supplement: Supplementary file 6 — Supplementary Figure 5 [file 41419_2019_1682_MOESM6_ESM.pdf]
